# Body and mind: retention in antiretroviral treatment care is improved by mental health training of care providers in Ethiopia

**DOI:** 10.1186/s12889-018-5821-y

**Published:** 2018-07-20

**Authors:** Tezera Moshago Berheto, Sven Gudmund Hinderaker, Mbazi Senkoro, Hannock Tweya, Tekalign Deressa, Yimam Getaneh, Gulilat Gezahegn

**Affiliations:** 1 Ethiopian Public Health Institute, HIV/AIDS and TB Research Directorate, P.o.box 138, Wolaita Sodo Addis Ababa, Ethiopia; 2 Centre for international Health, University of Bergen, Bergen, Norway; 3 National Institute for Medical Research, Muhimbili Medical Research Centre, Dare Selam, Tanzania; 4 Medecins Sans Frontieres, Medical Department (Operational Research) Brussels Operational Center, Luxembourg, Luxembourg; 5 Guraghe Zonal Health Department, Disease Prevention and Control Unit, Wolkite, Ethiopia

**Keywords:** ART, Mental health care, Retention on care

## Abstract

**Background:**

Ethiopia has achieved a high coverage of antiretroviral treatment (ART), but maintaining lifelong care is still a great challenge. Mental illnesses often co-exist with HIV/AIDS and may compromise the retention on ART. In order to improve prolonged retention in ART care, basic training in mental health care was introduced for ART providers, but this hasn’t been evaluated yet. The aim of this study was to examine if this training has improved patient retention in care.

**Method:**

A retrospective cohort study was employed to compare attrition from ART between clients attended by care provider trained with basic mental health service (exposed) and those in the standard ART follow-up care (unexposed) in public health facilities. A routine patient follow-up electronic database enrolled for ART between 2005 and 2017 was abstracted for the study. The Kaplan-Meier plot was used to compare the attrition rates between the two groups. The log-rank test was used to assess differences in the groups. The Cox proportional hazards regression model was used to determine predictors of attrition. We used estimated effect size of hazard ratios (HR) with 95% confidence intervals (CI).

**Result:**

During the 12 years of observation, 8009 study participants under ART were followed for 33,498 person-years. The incidence of attrition was 6.5 per 100 person-years and 21% higher in the unexposed group (HR 1.21; 95% CI 1.1, 1.3), and retention in care was significantly higher in the mental health exposed group throughout the study period. WHO clinical staging III/IV, tuberculosis coinfection, the male gender, and poor functional status were independent risk factors for attrition.

**Conclusion:**

We found that clients in the group exposed to mental health care training tended to have better retention in ART care with some variation according to gender, WHO Clinical stage and functional status. Training of ART providers in mental health may be considered in order to strengthen ART retention in low resource settings.

## Background

Generally, mental health problem and HIV/AIDS coexist with a complex bidirectional interaction [1–3]. Globally, mental health problems are more than twice as common among people living with HIV/AIDS (PLWH) as the general population [4, 5]. There are several possible explanations for the higher prevalence of mental health problems among PLWH. First, mental health problems such as depressive symptoms have been reported to be associated with HIV disease progression [6] and treatment outcome [7]. Second, mental health problems could be a manifestation of a direct effect of HIV on the brain cells [8, 9]. Third, the strains of living with HIV/AIDS, as well as problems with poor sleep and chronic pain caused or aggravated by the illness, can also cause mental health problems [9]. Furthermore, beyond the disease process and the chronic conditions, the stigma and discrimination attached to this infection have been shown to augment the mental health problem [2, 9–11].

In HIV patients, mental health problem can be a significant barrier to health behaviors such as retention in medical care and ART adherence, and thus pose a critical challenge for HIV care and control program [5, 7, 10]. A study of HIV-infected patients in Uganda, for example, revealed that 47% (95% CI: 39–55%) of patients with a mental health problem remained in care 12 months after ART initiation, compared to 65% (95% CI: 61–69%) of those without mental illness [10]. On the other hand, mental health support by providers has been reported to improve both retention in care and adherence among HIV patients [5, 7]. Therefore, mental health-related problems for HIV/AIDS patients who initiate ART may benefit from addressing such issues. However, in many countries including Ethiopia, this has not been properly recognized.

Ethiopia is one of the least developed countries in Africa that is severely affected by HIV/AIDS. The population in need of ART was estimated to be more than 770,000 in 2017 [11]. To ensure the accessibility of the care, the service has been decentralized to primary health care level since 2006 [11]. As a result, the service is available for a large part of the population in need. Furthermore, the country has launched the three 90s treatment target for HIV/AIDS prevention and control by 2020: 90% to know their HIV status, 90% of diagnosed HIV patients to receive ART, and 90% of HIV patients on ART to have viral suppression. Currently, high attrition rate, poor adherence to treatment regimen, and early mortality are prominent challenges of the HIV/AIDS program in the country. Recent studies in Ethiopia indicated attrition to be around 20% at 6 months of follow-up [12, 13], which could foster poor treatment outcomes, emergence of drug resistance and potential transmission of drug-resistant virus strain.

Mental illness can aggravate adverse outcomes of ART [14, 15], and integration of mental health care into ART clinics was found to be effective in improving the ART outcomes [9]. Provision of ART has previously not focused much on the impact of mental illness, but the 2016 WHO consolidated guidelines for ART includes a section on treatment of mental health problems such as depression in clients on ART care packages. In Ethiopia, integration of mental health care into the ART clinics through the provision of basic training of mental health care for ART care providers has been implemented stepwise since 2005. However, the effectiveness of these training has not been evaluated.

Hence, the present study is aimed to determine the effect of basic mental health care training of ART providers on the outcome of ART in Ethiopian public health facilities.

## Methods

### Study design

A retrospective cohort study was employed to compare outcomes of ART between two different therapeutic approaches; care provider trained with basic mental health care and the standard ART follow-up care.

### Setting

The study was conducted in Southern Nations, Nationalities and People regional states (SNNPRS) public health facilities providing ART service. This region is located in Southern parts of Ethiopia. It is the third most populous regional state in Ethiopia accounts for 20% of an estimated 103 million people. The region is administratively divided into 15 zones, three special districts, and one city administration. The Ministry of Health of Ethiopia follows a three-tier healthcare system, including a few private health care facilities concentrated in urban areas. The ART is provided free of direct cost mainly in public health facilities, but also in selected private ones. The ART program is directed centrally from the ministry of health, all the health facilities in the country follow similar modalities and national guidelines, which are developed based on the WHO guidelines. Usually, the ART clients visit health care facilities bimonthly in the early phase of the care and then every two to three months appointment based on the client’s adjustment to treatment adherence. On-demand visit is always open if the need arises from the client side. The National Mental Health Strategy is expanding the integration of mental health services into HIV follow-up clinic throughout the country through training of basic mental health care for the non-specialist care provider, with the first training in 2005.

In the SNNPRS two zones were selected to study based on similar sociodemographic characteristics, setting, and the level and timing of initiating the training of the basic mental health care. All the public health facilities were categorized as “exposed” or “not exposed” to mental health training by a panel of experts in the health departments, based on their knowledge of plans, processes, and progress. Criteria for categorizing as “exposed” facility were whether ART providers received basic mental health care training. Patients who had moved from a non-exposed facility to an exposed or vice versa were excluded. Out of 25 public facilities, we selected purposefully three exposed and five unexposed facilities. ART care providing facilities with care providers recently trained with basic mental health care training and/or interrupted service due to staff turnover and failure to sustain the integrated service were excluded.

### Specific settings

The study was conducted in seven health facilities: three exposed facilities (Bue Health Center, Butajira Hospital and Butajira Health center); and four facilities (Sodo Health center, Sodo General Hospital, Atat Hospital and Wilkeite Health Center) where the care providers were unexposed. The facilities in the exposed part are supported by the PRIME—Ethiopia project of Addis Ababa University, Department of psychiatry by training the care providers.

### Study population

The study population included all HIV infected people enrolled for ART care between January 2005 and May 2017 in public health facilities in the study area.

### Description of the mental health care intervention package

**Fig. 1:**
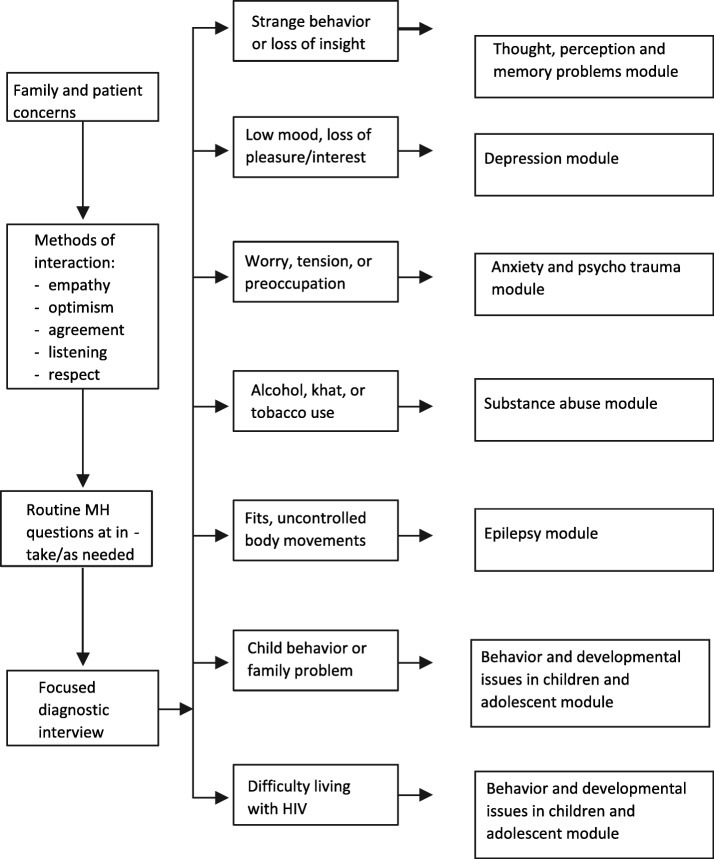
Flowchart for generic classification of mental problems and training plan. (Source: Detecting and managing common child and adult mental health problems in HIV care training package participants manual, second edition 2010, FMOH, Addis Ababa, Ethiopia)

ART care providers (nurses, health officers, physicians, and psychologist) in the integrated facility have received a seven days additional basic mental health care training to diagnose mental health problems and either to treat patients with basic psychotropic medications or refer them to an area hospital that provides psychiatric services. The overall goals of the training are (1) to enable primary health providers to work together in teams to recognize mental health problems among individuals receiving HIV care, and to deliver brief, first line interventions; (2) to enable primary health providers to collaborate with mental health specialists (nurses, psychologists, social workers, and psychiatrists) in the evaluation and treatment of mental health problems among individuals receiving HIV care; (3) to be familiar with the many different ways in which people with mental health problems can be helped; and (4) to be familiar with “stepped” approaches based on response to initial treatment and follow-up. The training includes the following topics: communication skills and assessment, thought, perception, memory problems, depression, anxiety, psychotrauma, substance abuse, epilepsy, behavior and developmental issues in children and adolescents, mental health aspects of living with HIV, and implementation issues (Fig.1 ).

The identified psychotropic medications from the essential drug list and targeted conditions were: haloperidol, chlorpromazine amitriptyline, fluoxetine diazepam, bromazepam, phenobarbital, phenytoin, carbamazepine. Memory loss/dementia is treated with ARVs, and substance abuse is treated with counseling. According to the Sodo district health department, all ART care providers were trained during 2005 and 2006 fiscal years, and it’s being given to new recruits continuously. The ART regimens used by the program are standardized [11].

### Data variables, sources of data and data collection

#### Data

We used the data collected routinely in HIV/AIDS clinic for patient monitoring and evaluation purposes. The main outcome variable was attrition a combination of LTFU, death and discontinued. As determinants of retention in ART, we assessed age, gender, TB-co infection, CD4 cell count, WHO clinical stage, INH prophylaxis, Cotrimoxazole supplement, provision of mental health service, adverse drug reaction.

### Definitions of variables

The following operational definitions were used in this study. *Lost to follow-up (LTFU)*: clients who missed scheduled clinical appointments for more than 90 days in a calendar year. *Retained in care*: clients who were on ART at the end of the study follow-up. *Attrition*: Clients who were recorded as LTFU, discontinued the ART, died after initiation of the ART care within the study period. *Discontinued*: clients known to have abrupted ART for any clinical or personal reason. *Died*: a patient who died after initiation of the ART regardless of the causes of death.

### Analysis and statistics

The data were routinely collected in the facility for clinical monitoring and evaluation, and entered into the national ART registration database in each facility with a unique identification number. Health personnel working in the clinic recorded all the data. Data recording started from the date that patients started regular HIV care in the clinic to confirmation of the final event. The database recorded was exported into Microsoft Excel, checked for inconsistencies and completeness, and then cleaned. Statistical analysis was done using R 3.4.2 (Comprehensive R Archive Network) and Stata Statistical Software (Release 14. College Station, TX: StataCorp LP).

The time to attrition was calculated in months according to the time interval between the dates of ART initiation to the occurrence of the event of interest (death, loss to follow-up or discontinuation), as recorded by the ART registration personnel. Patient characteristics were described in terms of mean, median or percentage, as appropriate. In order to account for changes in eligibility criteria for protocols of ART initiation to the year of the major change effected was used for analytic adjustments. Kaplan-Meier plots were used to compare attrition rates between “exposed” and “unexposed” clients. The log-rank test was used to compare the two groups. Multivariable Cox proportional hazards regression models with clustered robust estimates to account for an inter health-facility correlation were used to identify the independent effect of mental health service and other predictors of attrition. We used estimated effect size of hazard ratios (HR) with 95% confidence intervals (CI).

## Results

Between 2005 and 2017, a total of 8009 study participants under ART were followed. Of them, 1854 (23.1%) were followed by providers who exposed to mental health services. About 59.7% (1107) of patients followed by the exposed group were women. They were followed for 33,498-person-years. The median follow-up duration was 42 with interquartile range (IQR) of 13 to 85 months for mental health service exposed and 38 (IQR 11, 85) months for unexposed.

The median age of the participants in exposed groups was 31 (IQR 25, 38) years and 33 (IQR 24, 39) years for un-exposed. The median CD4 count at the start of ART was 171 (IQR 87, 261cells/mm^3^) among exposed and 172 (IQR 93, 272) among unexposed. TB-HIV co-infection was present in 387 (20.9%) of the exposed and 1017 (16.5%) of unexposed participants in the follow-up period.

By the end of the follow-up, excluding the patients transferred to another place, 968 (52%) of exposed clients and 2753 (44.7%) of unexposed were retained in care. Treatment outcomes reported were as follows in the exposed versus (vs) the unexposed groups: died 297 (16%) vs 550 (8.9%), drop out 140 (7.6%) vs1125 (18.3%) and transfer out 438 (23.6%) vs 1648 (26.8%). There were no statistically significant differences between the two groups in baseline demographic, ART regimen (including efavirenz) and CD4 cell count. The regimen was changed in 528 (28.5%) of the exposed participant, and 1652 (26.8%) of the unexposed participants (Table 1).

**Table 1:** Baseline and follow-up characteristics of participants under ART with mental health trained and non-trained providers, Ethiopia

Characteristics	*Facility category*		
	Exposed n (%)	Unexposed n (%)	*P* value
ART Enrolment year			
2005–2007	408 (22.1)	1569 (25.6)	0.08
2008–2010	639 (34.6)	2179 (35.5)	
2011–2013	433 (23.4)	1244 (20.3)	
2014–2017	369 (20.0)	1138 (18.6)	
Gender			
Male	747 (40.3)	2602 (42.3)	0.13
Female	1107 (59.7)	3553 (57.7)	
total	1854 (23.1)	6155 (76.9)	
WHO stage at entry			
Stage I	408 (24.3)	1272 (20.9)	0.01
Stage II	348 (19.1)	1592 (26.1)	
Stage III	866 (47.5)	2600 (42.6)	
Stage IV	203 (11.1)	634 (10.4)	
Functional status at entry			
Bedridden	130 (7.3)	353 (5.9)	0.07
Ambulatory	580 (32.5)	1515 (25.3)	
Working	1072 (60.2)	4116 (68.8)	
TB Coinfection			
No (−ve)	1467 (79.1)	5138 (83.5)	0.01
Yes (+ve)	387 (20.9)	1017 (16.5)	
INH prophylaxis			
Yes	865 (46.7)	3222 (52.3)	0.01
No	989 (53.3)	2933 (47.7)	
Ever Regimen Substitution			
No	1326 (71.5)	4503 (73.2)	0.23
Yes	528 (28.5)	1652 (26.8)	
Ever Regimen Switch			
No	1819 (98.1)	6101 (99.1)	0.01
Yes	35 (1.9)	54 (0.9)	
Viral load Test (*n* = 1512)	270	1242	
Detectable	41 (15.2)	305 (24.6)	0.01
Not Detectable	229 (84.8)	937 (75.4)	

The overall incidence rate of attrition was 6.5 per 100 person-years and was higher in unexposed than exposed (HR 1.21; 95% CI 1.1, 1.3). The overall attrition rate from ART gradually declined with duration of treatment. The probability of retention (95% CIs) on ART at the 6th, 12th, and 24th months is shown in Table 2.

**Table 2:** Retention in ART care during follow-up attending exposed and unexposed ART providers in Ethiopia

Follow-up time in month				
	Cumulative probability (95% CI) of retention in care after attrition from deaths		Cumulative probability (95% CI) of retention in care after any attrition	
	Exposed	Unexposed	Exposed	Unexposed
6	94.5 (93.4, 95.5)	95.4 (94.8, 95.9)	92.4 (91.1, 93.6)	90.9 (90.1, 91.6)
12	92.1 (90.8, 93.3)	93.5 (92.8, 94.1)	88.1 (86.4, 89.5)	84.5 (83.6, 85.4)
18	90.6 (89.1, 91.9)	93.0 (92.3, 93.7)	86.0 (84.3, 87.6)	81.2 (80.1, 82.2)
24	89.6 (88, 90.9)	92.6 (91.86, 93.3	84.3 (82.5, 86.0)	79.1 (78.0, 80.1)
48	82.9 (80.8, 84.8)	90.8 (89.93, 91.6)	76.9 (74.6, 78.9)	73.3 (72.0, 74.5)
144	73.2 (68.4, 77.4)	81.3 (77.5, 84.6)	63.3 (58.6, 67.6)	52.6 (47.3, 57.5)

*CI* Confidence interval); *Exposed* Exposed to mental health care

**Fig. 2:**
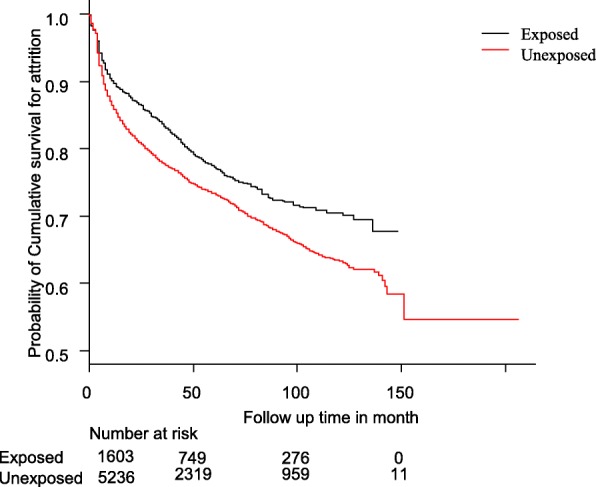
Kaplan-Meir estimates of attrition by exposure status for mental health care adjusted for age, baseline CD4 count, baseline functional status and WHO clinical stage at entry

The Kaplan Meier plots (Fig. 2) and (Table 2) revealed that clients exposed to mental health service had higher overall retention in care than the unexposed, but the number of a verified death was higher in the exposed group.

### Factors associated with the attrition from ART

Controlling for other demographic and clinical characteristics in a Cox-proportional hazard regression analysis, mental health service exposure had significantly increased retention in care, and the risk of attrition in the unexposed group was 20% higher than the exposed group (HR 1.2; 95% CI 1.1–1.4). Furthermore, age, advanced WHO clinical staging, not using cotrimoxazole prophylaxis, TB-coinfection, not getting isoniazid prophylaxis, male gender, and functional status were independent risk factors for outcomes of ART (Table 3).

**Table 3:** Analysis of factors associated with attrition among HIV infected patients Under ART in South Ethiopia

Variables	Attrition n (%)	AHR (95% CI)
Mental health		
Unexposed	1750 (79.6)	1.2 (1.1, 1.4)
Exposed	1063 (48.4)	Ref
Age (IQR)	31.75 (11.13)	0.9 (0.99, 1.0)
BaseCD4count (IQR)	220.51 (760.59)	0.9 (0.95, 1.0)
Baseline WHO clinical		
WHO stage [I]	298 (13.9)	Ref
WHO stage [II]	421 (19.6)	1.1 (0.9, 1.3)
WHO stage [III]	1042 (48.5)	1.2 (1.01, 1.4)
WHO stage [IV]	386 (18)	1.5 (1.2, 1.8)
CTM prophylaxis		
(No)	314 (14.3)	1.2 (1.01, 1.3)
Yes	1884 (85.7)	Ref
INH prophylaxis	445 (20.2)	0.2 (0.14, 0.2)
(Yes)		
No	1753 (79.8)	Ref
TB Coinfection		
(Yes)	455 (20.7)	Ref
No	1743 (79.3)	0.7 (0.6, 0.7)
Gender		
Female	1135 (51.6)	Ref
Male	1063 (48.4)	1.2 (1.1, 1.3)
LowestCD4 (IQR)	164.05 (163.07)	0.9 (0.9, 0.99)
Regimen Substitution (No)	1845 (83.9)	0.14 (0.12, 0.2)
Substitution (yes)	353 (16.1)	Ref
Baseline functional status		
Working	1135 (51.6)	0.6 (0.5, 0.7)
Ambulatory	726 (33.0)	0.5 (0.4, 0.5)
Bedridden	280 (12.7)	Ref
ART Enrolment year		
2005–2007	610 (28)	Ref
2008–2010	830 (38.2)	1.22 (1.1, 1.36)
2011–2013	435 (20)	1.5 (1.31, 1.73)
2014–2017	300 (13.8)	2.3 (1.96 2.75)

‘Analyzed by Cox proportional hazard regression, with adjusted hazard ratio (AHR) with 95% confidence interval

## Discussion

This study confirmed the hypothesis that basic mental health care service training for ART care providers improved patient retention in care. Patient under ART from facilities with ART providers trained on basic mental health care tended to be retained longer. The risk of attrition from the ART care was 20% higher in facilities unexposed to mental health services as compared to facilities exposed to mental health care. A considerable proportion of the attrition occurred in the first six months of ART initiation.

Proper patient/provider relationships and clinic experiences are known to improve patients’ trust on care providers and can significantly impact retention and adherence of patients. Therefore, the overall better retention of clients observed in the exposed group could be a result of a better counseling of patients on drug adherence, coping mechanisms with drug adverse effects, and mental health problems such as depression and anxiety which may jeopardize the success of ART. The effect may be partly due to the training of ART providers in mental health packages.

Consistent with our findings, studies indicated that behavioral intervention, which is considered as a part of the counseling package, had improved the adherence to ART treatment, viral suppression, and an overall outcome of ART. Furthermore, a meta-analysis that evaluated the efficacy of behavioral interventions for alcohol abuse, a major health problem among PLWH, indicated a significant improvement in HIV-related health outcomes [16–18]. Considering these, our finding has important implications for current ART care in Ethiopia and underscores the need for integration of mental health care into ART clinics to improve treatment outcomes.

The overall Kaplan-Meir estimates of retention show that the largest attrition occurred in the first year. The probability of retention in the unexposed group was comparable with other studies conducted in Ethiopia [12, 13]. Retention in care in the exposed groups from the present study seem to be higher than other studies in a similar setting unexposed for mental health care [13, 19].

The causes of attrition in the present study were significantly different between the two groups. In the exposed group, attrition was mainly from verified death whereas LTFU was found to be the major cause for attrition in the unexposed group. The later was in agreement with the existing evidence [12, 13, 20]. Different studies indicated that in low resource setting with poor vital registration system like in Ethiopia, deaths, and causes of death are not reported. A meta-analysis of the LTFU patients in Africa revealed that 12 to 87% were due to death [21]. Thus, most of the LTFU clients would have died but pass unnoticed and are categorized incorrectly as a loss-to-follow-up. Due to a possible enhanced client counseling and better communication, it is possible that the probability of identifying the true outcome of LTFU in the exposed group might be higher than in the unexposed group. However, more rigorous research is needed to establish the relationship between the basic training of mental health care for PLHIV and the improvement in the outcome of ART.

In this study, we found that male gender has 20% higher attrition rate as compared with women. Some reports find no significant difference [12], but the majority of the existing evidence indicates that in ART women have better outcomes than men [13, 21, 22]. This could be due to several reasons, including different lifestyle between the two genders; males use more alcohol; and different coping thresholds. This study also found that subjects with a history of TB-coinfection were at higher risk for attrition from ART. This is consistent with other studies [12, 22]. TB is a known coinfection and major causes of AIDS-related death. ART client not getting INH prophylaxis, not using CTM prophylaxis were associated with better experiences of retention in care. This may be confounded by the eligibility criteria for the prophylaxis. People highly vulnerable to opportunistic infection are usually eligible for the prophylaxis.

Bed ridden and ambulatory patients were at higher risk for attrition from ART. In line with this data, evidences indicate that a baseline advanced disease progression was associated with higher risk of attrition [23, 24]. These suggest that being at poor clinical condition could be the contributing factor for the higher attrition rate.

### Strength and limitation

The strength of this study was the large sample size and the long duration in both mental health services exposed and unexposed groups. Another strength is that this is “real life” situation reflecting realities in public health in the districts studied. We also think that this may have a positive impact on the awareness of mental health and their often-neglected services in the country.

One limitation of the study is that we did not have any measurement of the level of mental health care training. Thus, the effect of this training on the magnitude of mental health disorders deserves a separate study. Furthermore, this study analysed the retrospective records of patients; and the accuracy of this data depends on the data collection and recording systems of the facilities. Some misclassification of exposure to MH training is obviously possible, with consequences for the results; however, it is probably minimal as the classification was done by individuals responsible and close to the program with a likely correct appraisal.

## Conclusion

In conclusion, better retention in care was found among the clients of ART care providers who got basic mental health care training. However, there was some variation according to gender, baseline progress of the disease WHO Clinical stage and functional status. Training of ART providers in mental health may be considered in order to improve ART outcomes in low resource settings.

## Abbreviations

AHR: Adjusted hazard ratio
AIDS: Acquired immune deficiency syndrome
ART: Antiretroviral treatment
CTM: Cotrimoxazole
HIV: Human immunodeficiency virus
LTFU: Loss to follow-up
PLHIV: People living with HIV
WHO: World Health Organization
